# Wireless Link Selection Methods for Maritime Communication Access Networks—A Deep Learning Approach

**DOI:** 10.3390/s23010400

**Published:** 2022-12-30

**Authors:** Michal Hoeft, Krzysztof Gierlowski, Jozef Wozniak

**Affiliations:** Faculty of Electronics, Telecommunications and Informatics, Gdansk University of Technology, G. Narutowicza 11/12, 80-233 Gdansk, Poland

**Keywords:** maritime communication, deep learning, wireless heterogeneous networks

## Abstract

In recent years, we have been witnessing a growing interest in the subject of communication at sea. One of the promising solutions to enable widespread access to data transmission capabilities in coastal waters is the possibility of employing an on-shore wireless access infrastructure. However, such an infrastructure is a heterogeneous one, managed by many independent operators and utilizing a number of different communication technologies. If a moving sea vessel is to maintain a reliable communication within such a system, it needs to employ a set of network mechanisms dedicated for this purpose. In this paper, we provide a short overview of such requirements and overall characteristics of maritime communication, but our main focus is on the link selection procedure—an element of critical importance for the process of changing the device/system which the mobile vessel uses to retain communication with on-shore networks. The paper presents the concept of employing deep neural networks for the purpose of link selection. The proposed methods have been verified using propagation models dedicated to realistically represent the environment of maritime communications and compared to a number of currently popular solutions. The results of evaluation indicate a significant gain in both accuracy of predictions and reduction of the amount of test traffic which needs to be generated for measurements.

## 1. Introduction

The e-navigation concept, as defined by the IMO (International Maritime Organization) in 2005 [1], brings a significant change of approach to maritime IT services, both allowing a wide set of communication technologies to be employed for the traditional maritime tasks and accelerating development of completely new services—including non-critical ones (e.g., aimed to improve comfort of crews). Loosening a traditional approach of specifying a particular communication technology to be used to provide a given maritime service, resulted in a growing interest in the subject of providing a digital communication at sea. Analysis of proposals for various maritime services (Maritime Service Portfolios, MSPs) [2,3], shows that there is a place for deployment of a diverse range of communication technologies, starting with global satellite systems through medium and long range terrestrial radio systems operating in medium (MF), high (HF) and very high frequencies (VHF) and ending with short-range, high-frequency broadband solutions. A current practice shows that long-range systems (MF, HF, VHF) are most commonly used for established, safety-related services, while short-rage broadband solutions are intended to support new MSPs related to, for example, health, logistics, administration, technical support and well-being of crews.

A significant part of current e-navigation research and development activities is focused on improving communication in specific types of sea areas (e.g., areas of a high maritime traffic density) or is related to deployment scenarios, such as these, where a reliable and efficient communication is required within a group of vessels (e.g., fishing boats on an area allowing communication between them [4,5]). Resulting solutions often employ two- or multi-hop communication between vessels at sea or between such vessels and onshore stations, and can be classed as self-organizing mesh networks. A number of pilot projects implementing such maritime communication solutions have been undertaken [6,7,8]. Their activities either focus on theoretical approach to various aspects of communication (such as radio transmission or routing algorithms [4,9]), or take a practical approach, aiming to create solutions ready to be implemented in specific scenarios [4,10,11,12].

Despite the above-mentioned research and development activities there is an area of maritime communication that, despite its importance, is relatively rarely specifically addressed by such (either theoretical or practical) activities—it is a maritime communication in coastal areas. The cause for a relatively limited interest is the fact, that there already is a number of technologies which can be employed to provide digital communication there using on-shore infrastructure, including public, on-shore subscriber networks [13]. Such state of affairs tend to suggest, that the problem of providing maritime access to on-shore networks has already been solved in coastal areas—which, as we are going to illustrate in this paper, is not the case. At the same time, coastal areas are of high importance for maritime operations. With their high shipping density, critical sub-areas (such as port approaches), multiple types of possible maritime activities, diverse range of requested services (including, e.g., support for port-related logistical, organizational and legal operations) and complex navigational problems, coastal areas set high requirements for the availability and quality of digital maritime communication.

On-shore subscriber networks cover most well-developed land areas and offer network access using a number of different wireless technologies. The multitude of well-defined telecommunications standards allows creation of heterogeneous access networks which permit users to choose from a number of alternative communication technologies depending on their Quality of Service (QoS) requirements and current communication situation. Such heterogeneous network environments enable a flexibility in adapting to a number of individually defined requirements and offering optimized set of services to end users. At the same time, such access systems employed on-shore are almost exclusively based on a stationary infrastructure deployed by a network operator, providing network coverage over a specific area, located in direct neighborhood of access network infrastructure elements. This approach makes them of little use for general maritime employment, but still useful for coastal communication where it is possible to provide coverage of off-shore area using infrastructure located near shoreline.

With typical, popular access systems employing diverse general-purpose solutions such as Wi-Fi (Wireless Fidelity), 4G-LTE (Long Term Evolution) or WiMAX (Worldwide Interoperability for Microwave Access), as well as some purpose-designed systems (such as [14] the coastal communication environment will invariably be a heterogeneous one. Moreover, the constantly growing demand for higher throughput with the simultaneous guarantees of connection stability, causes network operators to constantly update and modify their systems, adapting new technologies to address the needs of subscribers. Such a presence of multiple access networks seems to create an environment facilitating maritime communication, but it also makes the problem of selecting the best means of communication to be employed by a vessel in a given moment to be one of critical importance—even more so, because we can expect available access networks to employ different transmission technologies offering distinct communication characteristics.

Despite these challenges, we are convinced that the use of heterogeneous wireless networks for maritime communications can bring significant benefits in terms of increasing the efficiency and availability of such communications—especially in coastal areas, where a diverse, land-based access infrastructure can be used to provide coverage for maritime vessels. However, the practical use of such systems presents a number of challenges, among them the need to select the link that offers the best performance in a particular situation.

In this paper, we focus on the problem of selecting a best available communication link from a set of possibilities offered by a heterogeneous communication environment with multiple communication technologies available to vessels using shipping lanes or waiting at port approaches and thus having access to on-shore communication infrastructure. As review of literature on the subject have not provided a satisfactory answer, we propose our own solutions employing deep learning mechanisms—aimed to provide an accurate indication of the link capable of providing the highest TCP throughput, while minimizing the impact of the assessment process on available communication resources.

Main contributions of the paper include:

- an overview of handover procedures and important characteristics of maritime wireless access systems, necessary for reliable modelling of such communication environment,
- the use of deep learning methods to significantly reduce the measurement traffic required to perform the link assessment procedure in the marine environment,
- an evaluation of the proposed methods in comparison with other, commonly employed solutions.

The paper is organized as follows. Section 2 provides a description of existing works related to mobility management problems in access networks. Selected, popular methods of link quality assessment and selection are presented in Section 3. As wireless signal propagation over sea areas differs significantly from its propagation over land, specifics of the process (important from the perspective of data collection and learning algorithms) are presented in Section 4. Proposed solutions to link selection problem and their evaluation methodology are described in Section 5. This section, together with Section 6, in which system modelling and evaluation are presented, form a description of the methodology applied in our research. Limitation of the proposed methods, as well as interesting directions for future works are discussed in Section 7. The paper is concluded in Section 8, confirming possibility of link-selection efficiency improvement achieved by means of proposed deep-learning solutions.

## 2. Mobility Management and Link Selection Procedure

From the brief description presented in the previous section, it is clear that maintaining network connectivity by a moving ship will require periodic changes of its point of network attachment (handover), often associated with a change in the access technology used (inter-technology handover). The process of preparing and conducting a handover requires three tasks to be executed: (i) information gathering, (ii) handover decision and (iii) handover execution. In our specific case, due to probable availability of multiple access systems, we can expect that an important part of handover decision phase will be a selection of a specific link to be used for data transmission from a set of link options available in a given circumstances [15].

### 2.1. Information Gathering Phase

The goal of the information gathering phase is to create a knowledge base sufficient to form a basis for a decision of when to perform handover and which of available access networks should be selected to hand over to. The information gathering process should be conducted continuously or repeatedly, to keep the resulting knowledge base current for Handover Decision mechanisms to use.

In case of a homogeneous system, defined as a system utilizing a single wireless technology (e.g., only LTE or only Wi-Fi networks), all its access devices are similar and provide similar parameters to describe their configuration and state, so we are able to use passive methods in the information gathering phase. It is possible to use values of different parameters provided by access devices and describing a state of a physical (e.g., signal strength indicator) and media-access control (e.g., channel usage) layers to compare estimated quality of available communication links between mobile node (maritime vessel) and access infrastructure.

The situation is much more complicated in a heterogeneous network (it should be noted that in this work term heterogeneous is used to describe a multi-technology network—a wider sense than sometimes used in respect to an LTE network with different size of cells (standard eNodeB, pico eNodeB etc.)), where access devices employ different technologies and provide different sets of parameters to describe their state. Moreover, even if similar parameters can be obtained for different technologies, their impact on communication link quality can be different for each of them.

As the coastal maritime communication environment is a heterogeneous one, containing a number of very loosely integrated or completely independent access networks utilizing a number of different technologies, a passive observation of a static set of state parameters reported by devices is not practicable or effective—different methods of operation that different access technologies employ make parameter-based comparison difficult and most often inconclusive. In this situation, the most effective solution seems to be the use of active probing to allow a mobile node to obtain information about the communication quality which it can expect for each considered link.

### 2.2. Handover Decision Phase

Deciding when to perform a handover and the correct selection of a new access network are crucial if network connectivity is to be maintained by the mobile node. This decision is based on information available in the knowledge base, updated periodically during the information gathering phase.

Different methods can be used to make switching (handover) decisions, usually belonging to several of the following classes [16]:

- decision functions—a class containing both single and multiple criteria decision algorithms (Multiple Attribute Decision Making—MADM [17]) providing an ability to indicate a new network from a set of available networks with respect to different parameters (e.g., communication delay, jitter, bandwidth),
- fuzzy logic—a class of solutions (mainly enhancements of solutions of the previous group), containing mechanisms able to operate with imprecise data describing network parameters,
- Markov Decision Process—consists of mechanisms providing a formal way to describe a heterogeneous network from a point of view of a mobile node observing such environment and periodically making decision based on a reward function (commonly related to resulting network parameters or communication QoS) [18],
- reputation systems—a class of approaches aiming to share experience between mobile nodes to compute a reputation value describing preference for using different access networks [19,20],
- machine learning (ML)—solutions employing ML models (including deep learning), which are trained based on collected data to improve handover efficiency [21].

Due to its simplicity, it is a common approach to use a single criterion decision function taking into account only one low-level (e.g., physical) parameter, which can be obtained directly from network interfaces, to chose a new point of network access. A very popular example is a Strongest Signal First method which utilizes Received Signal Strength Indication (RSSI) value to find an access device from which the mobile node receives the strongest radio signal. However, as we already mentioned, such solutions are practicable only in homogeneous networks [22] because the parameter used in comparison must be available for all access devices and impact the quality of communication in an uniform manner for them all. In heterogeneous environments, with their diverse access technologies, such a low-level parameter that could be directly used for link quality assessment and selection is most often impossible to find due to vastly different communication methods.

In addition to a specific method which is employed to make the handover decision, handover mechanisms can also be differentiated based on a network element which performs this task. Solutions, in which a mobile node decides when and for which network handover should be executed belong to a host-based mobility management group. The main advantage of such an approach is the possibility for handover mechanisms to be aware of all specific end-user requirements when making a handover and the ability to provide a flexible solution tailored to specific needs of a particular user. On the other hand, a group of mechanisms in which network-side elements are responsible for preparing and initiating a handover (network-based mobility management), provides the ability to reduce the amount of signaling necessary to be performed over a wireless link during the handover execution phase—an ability of high importance in case of potentially unstable links with high data loss ratios.

A good example of a standardized approach to information gathering and making handover decisions in heterogeneous environment are Media Independent Handover (MIH) standards [23,24] proposed by the IEEE 802.21 Working Group. The solution concentrates on the Information Gathering Phase and introduces a Media Independent Information Service (MIIS) for that purpose. With the extended knowledge base provided by MIIS, different handover schemes (both network-based such as [25] and host-based like [26]) can be employed to make a handover decision and execute it.

### 2.3. Handover Execution Phase

When a new candidate access network is selected as a result of Handover Decision Phase and a decision to perform a handover is made, a Handover Execution Phase begins. That phase can be implemented in a number of different ways.

In the simplest case, when we connect to another access device in the same network, handover execution is limited to mechanisms implemented at data-link layer (L2 handover). Since no change of configuration at the network layer and above is required, it is a solution transparent to higher layers of the network protocol stack. An example of this type of handover is changing the currently used access point within the same Wi-Fi network.

A more complex variant of a handover mechanism is the one in which we want to connect to a different access system, because it requires us to modify configuration at the network layer level. The scope of required changes will depend on the specific scenario and may include changing of a network address (such as an Internet Protocol address) assigned to a network interface or changing a network interface used for data transmission [27]. A number of complex solutions have been developed to support this complicated handover scenario. The most common are Mobile IPv6 (MIPv6) [28] and Proxy Mobile IPv6 (PMIPv6) [29] protocols, often extended with a number of modifications to improve handover efficiency (e.g., [30,31]).

As handover is a disruptive occurrence from the point of view of network communication, a number of approaches to the process have been defined, aiming to mitigate its negative effects. In a reactive-handover approach the mobility management procedure is performed after disconnection from the current network, resulting in a significant communication disruption. An opposite approach is a proactive-handover, where the information exchange procedure is carried out between a mobile node and network devices to prepare the handover process before currently used wireless link is terminated. A specific variant of proactive handover is a soft handover, possible for mobile nodes equipped with multiple network interfaces, where a node creates a new link before terminating the one currently in use.

A practical example of a handover execution solution designed to operate in a heterogeneous, maritime wireless systems is proposed in [6], combining the Proxy Mobile IPv6 protocol and the soft handover approach [32].

## 3. Common Link Selection Solutions

The above analysis indicates, that in a heterogeneous coastal maritime communication environment (consisting of multiple access networks employing diverse access technologies and belonging to multiple operators), making handover decision using a knowledge base limited to a static set of network interface parameters would not bring satisfactory results. The problems with selecting a set of universally available parameters would be compounded by difficulty in successfully using them to predict the expected quality of a communication link between a mobile node and access infrastructure.

In this situation, there is a need for a higher-level parameter, which does not describe physical conditions subsequently used to predict communication quality, but rather one which describes the communication quality directly—for example a measured link transmission delay or packet error rate of an ISO-OSI network layer protocol (e.g., Internet Protocol—IP). Such a parameter, while directly providing information about quality of network communication, often requires an active measurement to be performed—for example, by generating test traffic of specific parameters and monitoring its handling by the network. The use of active measurement methods results in increased consumption of network resources as test traffic is periodically transmitted. This requirement can be a significant disadvantage in case of wireless links operating in difficult conditions. To minimize the negative impact of a testing method on the amount of network resources available to users, a care should be taken to chose a testing method minimizing the amount of the necessary test traffic.

There is also a theoretical possibility of employing user traffic for analysis of communication quality, without the need for devoting network resources for testing purposes. However, it is impractical to attempt to utilize the existing user traffic in such a manner. Due to the fact that servers providing services which users access are often located at a considerable network distance from the access network edge, such an attempt would introduce a difficult to predict influence of the on-shore network infrastructure (possibly maintained by many different operators). Additionally, response characteristics of the service (both normal processing requirements and events such as errors, overloads etc.) the user interacts with would also impact the measurements in an unpredictable manner. In this situation, the solution that brings the best results seems to be the use of test traffic generated for exchange through the link between the mobile node and the infrastructure of the access network.

The values of network layer parameter obtained by active testing can give us a credible assessment of the manner in which data packets will be delivered by a specific access link. However, both e-navigation and popular Internet services tend to employ additional transport protocols for their use. The most common is the Transmission Control Protocol (TCP), providing connection-oriented, reliable data delivery by means of retransmissions and incorporating congestion avoidance mechanisms limiting a transmission speed of a TCP source when a necessity to retransmit data packets occurs (as their loss is attributed to a network congestion).

Due to the fact, that transport layer mechanisms can significantly impact the resulting communication quality characteristics for services which employ them, we have decided to concentrate on transport layer communication quality as our priority when making handover decision. Moreover, we have chosen TCP protocol as our transport solution due to its popularity. For a number of years TCP traffic has been dominating the Internet, carrying about 80–90% of the volume [33,34] of its traffic. Although this state of affairs begins to change due to new solutions like QUIC (a connection-oriented protocol providing a stateful interaction between a client and server [35]), resent studies show that TCP is still the most widely used—currently 60% to 80% of traffic observed at IP Exchange points (IPX) uses TCP [36].

Apart from the above practical consideration, the selection of TCP for service-oriented link quality assessments is encouraged in the literature [37,38], as its dependence on such lower layer parameters such as packet loss, transmission delay and network-layer link throughput, makes it a good choice for assessing the overall quality of the link.

In subsequent subsections we have included descriptions of popular, active methods of testing network-layer communication quality. Their results are often used directly to make a handover decision by means of a simple (single parameter) decision function. It is our opinion, illustrated in a further part of the paper, that such approach can be significantly improved by employing more sophisticated decision algorithm, while still making use of the same, easy to employ, testing methods.

### 3.1. RTT Tests

A Round Trip Time (RTT) is one of the popular parameters describing communication conditions. A network RTT includes a time required to deliver the request from a client to a server and time necessary for the response from the server to reach the client, as well as time taken to process request and generate the response. When testing for RTT value by sending requests to a particular server, a service with a minimum and constant request processing time should be used, to retain the focus on network conditions and minimize impact of server performance and its current load. Because of this requirement, RTT tests are almost always performed actively, using a dedicated testing service, instead of relaying on passive observation of user traffic.

The value of RTT has a significant impact on performance of the TCP protocol, as the protocol employs a transmission window mechanism and requires acknowledgements of transmitted packets from the receiver, before the window can be moved to allow transmission of new packets [39]. In methods directly using RTT as a basis for the link selection, the link that is characterized by the lowest RTT value is selected to be used—see Equation (1) which describes this method:

(1) $$i_{s}=\underset{i}{\mathrm{argmin}}d_{i}\left(t_{o}\right)$$

where $t_{o}$ is an observation time, $d_{i}$ means round trip time for the *i*-th link and $i_{s}$ is the selected link.

While RTT testing is an active testing method, it can be employed using a very limited amount of test traffic, so its impact on the network system is minimal, which is a significant advantage of this method. However, not only the actual TCP throughput of a link but even link preference can be difficult to predict using exclusively RTT values, as other factors (such as packet errors) have a strong impact. Even if we take into account that in real deployments there often is a dependency between RTT and such other parameters it is strongly scenario-dependant and therefore difficult and unreliable to use.

### 3.2. PER Tests

Packet Error Rate (PER) metric shows a number of network packets lost in the relation to the total number of packets sent during the observation window ($t_{o}$). It is a simple metric, however, and does not provide information about grouping or bursts of packet losses, which have a significant impact on TCP operation [40,41].

However, even a simple PER value can provide a valuable information on the expected TCP throughput, as each loss causes TCP retransmission and reduction of the sending window size. When using PER value directly in the link selection process, in accordance with Equation (2), a link offering the lowest PER should be chosen. In the equation $p_{i}\left(t_{o}\right)$ refers to a PER value estimated over time $t_{o}$ for the *i*-th link.

(2) $$i_{s}=\underset{i}{\mathrm{argmin}}p_{i}\left(t_{o}\right)$$

The PER active testing method has an advantage similar to RTT test—both methods have a very small traffic footprint. In case of PER testing, it is even smaller, as it is possible to employ it using only unidirectional traffic. Moreover, as packet losses are of high importance for TCP performance, it can provide somewhat better predictions. However, as in case of the RTT, the precision of the TCP performance assessment based exclusively on PER is limited.

### 3.3. Active TCP Measurement

In this approach a test TCP transmission is conducted between a mobile node and a testing server to directly measure the link quality in respect of TCP performance. As shown by Equation (3), the TCP throughput metric ${\bar{T}}^{i}\left(t_{o}\right)$ can be defined as an average throughput measured during observation time $t_{o}$ for a new connection established over *i*-th link.

(3) $$i_{s}=\underset{i}{\mathrm{argmax}}{\bar{T}}_{i}\left(t_{o}\right)$$

As the measured value directly describes the quality (throughput) of TCP communication, the simplest decision method is to select for data transmission the link with the highest value.

This link evaluation method allows for a high accuracy of throughout assessment (as it is directly measured, not estimated), however it also generates a high volume of test traffic, consuming resources which could be used for actual communication. This issue may be especially important for small vessels or elements of remote sensing infrastructure with limited energy and communication resources.

Above-mentioned methods applied separately or combined by means of multiple-criteria decision-making solutions have been used for several years [16]. Although their use is still proposed in a number of recent papers, such methods have a number of well known limitations [42], which make their employment with simple decision methods not sufficient for services requiring a specific level of Quality of Service parameters such as delay or packet loss. Moreover, even multiple-criteria decision-making solutions are strongly dependant on weights assigned to different parameters used in making handover decision. Correct selection of weight values is often of primary importance for correct operation of such a solution, and tends to differ with different communication environments or scenarios.

A number of papers have been published addressing the above problem of communication link selection, taking different approaches to improve efficiency of the methods mentioned above.

In [43], a method for reliable link selection and data aggregation based on data aggregation factor (DAF) hase been proposed as a solution dedicated to wireless multimedia networks. Metrics such as transmission frequency, transmission energy, and link quality have been used in this method for selecting the optimal link set utilized by aggregation mechanisms. The results of simulation evaluation show that the proposed solution, in comparison with the existing mechanisms, allows the error rate to by reduced by 50% and the packet delivery ratio to be increased by 35%.

In [44], Funabiki et al. proposed a new active approach for access-point election based on link-rate changes in wireless IEEE 802.11n mesh networks. Their solution uses detailed network topology information with all nodes positions and measurements of throughput in a relation to distance between nodes.

In [45], Wang and Zhang described a machine-learning approach based on a decision tree intended to be used for network selection in heterogeneous wireless network. The authors proposed top-down greedy-type formal procedure for generating decision trees. Another machine learning solution is presented in [46] by Islam et al. The solution is based on reinforcement learning approach for link-channel selection problem in wireless mesh networks. Evaluation results performed by means of simulation experiments show increase of the aggregated network throughput up to 15%, the average end-to-end delay reduction as high as 12% and Jain’s fairness index value increase up to 10% when this solution is used to adjust link selection, channel allocation and power control at each forwarding node.

QoS-aware base-station selection solution intended for a distributed wireless MIMO downlink is proposed in [47]. It aims to reduce the base station utilization and to minimize the interfering range observed in distributed MIMO systems. The authors of this method conducted numerical simulations and their results confirmed that it outperforms baseline schemes.

Nomikos et al. proposed (in [48]) low-complexity algorithm for link selection aiming to reduce transmission delay and to increase efficiency of asymmetric two-hop wireless networks. Both centralized and distributed versions of this algorithm are described and evaluated in a numerical simulations that confirm the average packet delay reduction compared to other delay-aware algorithms.

An interesting game-theory approach to network selection and resource allocation in wireless access networks is presented in [49]. A model of noncooperative congestion game where players selfishly select a wireless access network aiming to minimize selection cost is analyzed in the paper.

The presented list of works related to the topic of the paper shows that while multiple approaches to the problem have been presented, the solutions are most often scenario and/or technology specific and as such lack the versatility. Moreover, all of the above solutions have been designed for the on-shore environment, which (as presented in the following section) is significantly different than the maritime one. Combined with their limited flexibility, their successful employment in maritime environment would be difficult.

On the other hand, promising solutions for versatile link quality evaluation take advantage of deep learning approach [38] and a number of the proposed solutions have been designed to provide a link-quality assessment. However, the existing methods were intended to be used as link quality degradation detection mechanisms, and cannot be expected to function efficiently as elements of a mobility management solution [50]. Furthermore, we should remember that deep learning approaches are operating by extracting knowledge from provided data sets, so a reliable data source allowing them to be trained to operate in a specific environment is of crucial importance for their effective operation. Further analysing this requirement, we concluded that there is a need to analyze specifics of the coastal maritime communication to pinpoint its essential aspects (which significantly differ from characteristics of on-shore systems).

## 4. Characteristics of the Wireless Maritime Communication Environment

At first glance, the marine communication environment seems to be simple, especially when compared to land-based urban environments, as there are no buildings and significant terrain features are encountered only sporadically. However, we should analyze the situation in more detail.

According to [51], the key factors when modelling propagation conditions at sea are:

- reflections,
- refraction,
- possibility of beyond line-of-sight transmission,
- parameters and locations of antennas and employed signal frequencies,
- humidity, including the existence of so-called evaporation channel.

In order to take the above factors into account when analyzing communications between ships and shore network infrastructure over relatively long distances, the following elements must be present in the employed propagation model:

- an appropriate path-loss model,
- a model introducing a diffraction of radio waves caused by sea surface,
- a vessel motions model.

**Figure 1:**
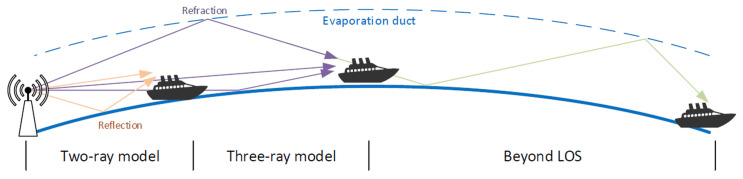
Difference between propagation models.

For our case of radio propagation over sea areas, different path-loss models may be applicable, depending on the distance between communicating devices [52]. We will take into consideration three such models of different complexity: a two-ray model, a three-ray model and a three-ray model extended to include a diffraction effect. The basic idea behind each of them is presented in the Figure 1.

The first one, the two-ray path-loss model, is well-known and often utilized in various environments due to its simplicity. It takes into account two paths of an electromagnetic wave—the main, direct one between the sender and the receiver and the second one, reflected from the ground or sea surface. However, this model is suitable only for short range communication and so its utility in our case is limited.

The second model—the three-ray path-loss model, additionally includes a third path of a radio signal, which exists due to refractivity of the atmosphere over the sea significantly changing with the altitude. This effect is caused by a significant presence of a water vapor near the sea surface [53] forming an evaporation duct (see Figure 1).

The inclusion of this important feature of the marine environment (evaporation duct) makes the three-beam model much preferable to the simple two-beam approach as it is able to provide useful path-loss prediction over a much wider range of communication distances (above $\frac{4\pi h_{t}h_{r}}{\lambda }$ [52]). Additionally, number of field experiments confirmed applicability of the three-ray path-loss model with real-world measurements [54,55,56].

The three-ray path-loss can be calculated using formulas presented as Equation (4) [52]:

(4) $$\begin{array}{cc} L(d,h_{t},h_{r},h_{e})= & \\ \left\{\begin{array}{cc} -10log_{10}\left(4{\left(\frac{\lambda }{4\pi d}\right)}^{2}{\left(sin\left(\frac{2h_{t}h_{r}\pi }{d\pi }\right)\right)}^{2}\right) & ,\mathrm{if}\;d\leq d_{breakout}\\ -10log_{10}\left(4{\left(\frac{\lambda }{4\pi d}\right)}^{2}{\left(1+△\right)}^{2}\right) & ,\mathrm{otherwise} \end{array}\right. \end{array}$$

$$\begin{array}{c} \mathrm{where}:\\ △=2sin\left(\frac{2\pi h_{t}h_{r}}{\lambda d}\right)sin\left(\frac{2\pi \left(h_{e}-h_{t}\right)\left(h_{e}-h_{r}\right)}{\lambda d}\right) \end{array}$$

and *d* is the distance between sender and a receiver, $h_{t}$ and $h_{r}$ are Tx and Rx antenna height, respectively, $h_{e}$ refers to effective height of evaporation duct, λ is the wavelength of the carrier frequency.

**Figure 2:**
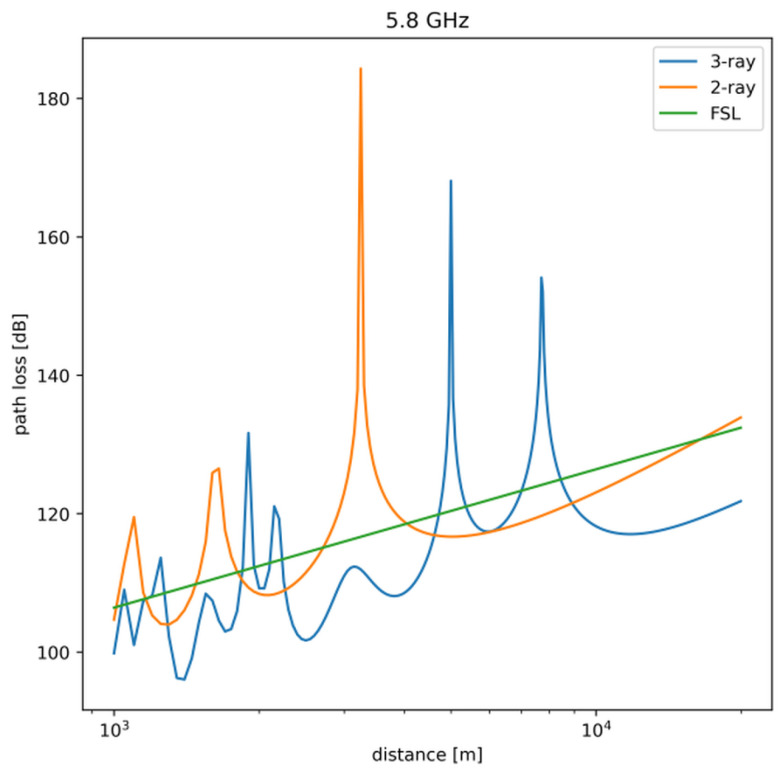
Propagation models comparison.

As can be observed in Figure 2, the differences in predictions provided by the more accurate three-ray model and simpler two-ray model are significant. The figure also includes results obtained by using the simplistic Free Space Loss (FSL) path-loss model.

However even the three-ray model requires extension, if it is to be used for long range communication scenarios. In such cases, an additional impact of an electromagnetic wave diffraction introduced by sea surface should be considered in accordance with ITU (International Telecommunication Union) recommendation P.526 [57]. The calculation of additional transmission loss due to diffraction over a spherical surface includes two additional parameters related to distance and antenna height. The relation between diffracted field strength *E* and the free-space field strength $E_{o}$ can be calculated as follows:

(5) $$\begin{array}{cc} 20log_{10}\frac{E}{E_{o}} & =T_{d}\left(X\right)+T_{ah}\left(Y_{t}\right)+T_{ah}\left(Y_{r}\right) \end{array}$$

where $T_{d}$ is the distance parameter and $T_{ah}$ is a antenna height. They are defined in accordance with Equations (6) and (7).

(6) $$\begin{array}{c} T_{d}\left(X\right)≅\left\{\begin{array}{cc} 11+10log_{10}\left(X\right)-17.6X & ,\mathrm{for}\;\;X\leq 1.6\\ -20log_{10}\left(X\right)-5.6488X^{1.425} & ,\mathrm{otherwise} \end{array}\right. \end{array}$$

(7) $$\begin{array}{c} T_{ah}\left(Y\right)≅\left\{\begin{array}{cc} 17.6\sqrt{\beta Y-1.1}-5log_{10}\left(\beta Y-1.1\right)-8 & ,\;\mathrm{if}\;\;Y\geq 2\\ 20log_{10}\left(\beta Y+{\left(\beta Y\right)}^{3}\right) & ,\mathrm{otherwise} \end{array}\right. \end{array}$$

where β is a parameter related to ground type and polarization. For over sea transmissions with horizontal polarization and frequencies above 300 MHz, in accordance with [57], β is assumed to be 1. *X* refers to normalized distance between sender and receiver, $Y_{t}$, $Y_{r}$ are normalized antenna heights, $a_{e}$ is the equivalent Earth’s radius. Normalization is performed using formulas presented in Equation (8).

(8) $$\begin{array}{cc} X & =d\beta {\left(\frac{\pi }{\lambda {a_{e}}^{2}}\right)}^{\frac{1}{3}}\\ Y_{t} & =h_{t}\beta {\left(\frac{\pi^{2}}{\lambda^{2}a_{e}}\right)}^{\frac{1}{3}}\\ Y_{r} & =h_{r}\beta {\left(\frac{\pi^{2}}{\lambda^{2}a_{e}}\right)}^{\frac{1}{3}} \end{array}$$

All of the models presented above are intended to be used for line-of-sight communication scenarios in which an unobstructed line of communication between sender and receiver exists, so they cannot be used for modelling beyond line-of-sight (BLOS) communication. For the purposes of our analysis it is sufficient, as BLOS communication requires precisely formed antenna beams [58] that would fit the trapping angle (lower that 1${}^{\circ }$)—an unrealistic requirement for general purpose, heterogeneous access infrastructure employed in civilian, coastal maritime communication. For this reason we consider BLOS transmission to be out of scope of this paper.

### Vessel Movements and Motions

The above overview shows that the correct selection of a path-loss model (and taking into account the diffraction effect) is an important element of modeling maritime communication. The use of simple models can alter the results in a significant way, making them of limited usefulness for real-word usage. In fact, the situation is even more complex, as we must bear in mind that the sea surface is generally not flat and is subject to a random movement. Movement of sea surface causes ships and buoys, and thus antennas installed on them, to move in various ways—specifically heave, sway, surge, yaw, roll and pitch. Such movement changes alignment of transmitter and receiver antennas, as well as their effective heights.

**Figure 3:**
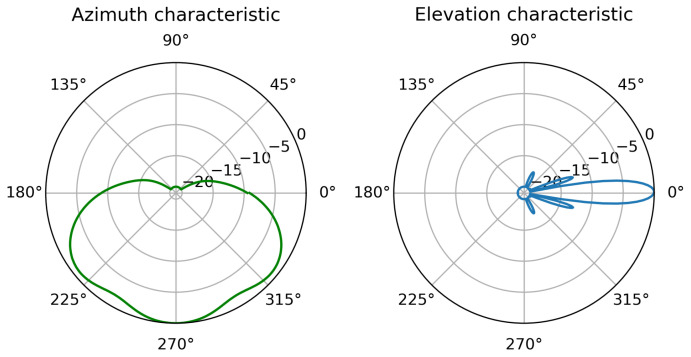
Mikrotik mANT19s antenna directional characteristics based on measurements provided by the manufacturer.

**Figure 4:**
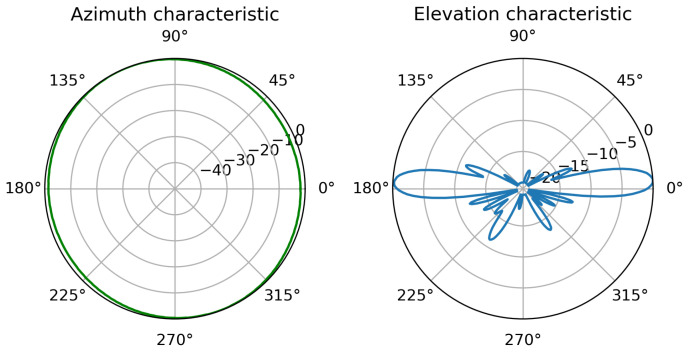
Ubiquity AMO-5G10 antenna directional characteristics based on measurements provided by the manufacturer.

The problem of changing alignment of onboard antennas is a highly important one as in maritime environment high-gain directional antennas with narrow beam width are commonly employed to overcome large path losses present due to long distances of communication. It is a frequent practice to employ sector antennas (such as Mikrotik mANT19s [59] with directional characteristics shown in Figure 3) at static installations (e.g., shore infrastructure, off-shore platforms, etc.), while mobile vessels employ omnidirectional antennas (such as Ubiquity AMO-5G10 [60], Figure 4). The narrow width of the vertical beam makes the signal strength variation caused by misalignment of antennas quite significant.

Additionally, the height at which antennas are located directly impacts the maximum range of line-of-sight communication and, as shown above, is an important parameter in many path-loss models—unpredictable changes of this parameter can have a significant impact on transmission quality as well.

The above observations clearly show the necessity of modeling spatial position changes of a vessel, if we aim to predict its real-world communication opportunities. Such position changes can be divided into two distinct types: vessel movement and vessel motion. The first one (movement) refers to changes of its geographic location and most often occurs due to intentional actions on part of the crew and is relatively simple to predict, as maritime vessels tend to follow administratively established shipping lanes and similar well-known paths. Moreover, additional real-world data can be obtained using Automatic Identification System (AIS) historical data [61]. Popular mobility models can be used to model vessels movement (e.g., Linear mobility model, Gauss–Markov mobility model, Turtle Mobility model) [62,63].

**Figure 5:**
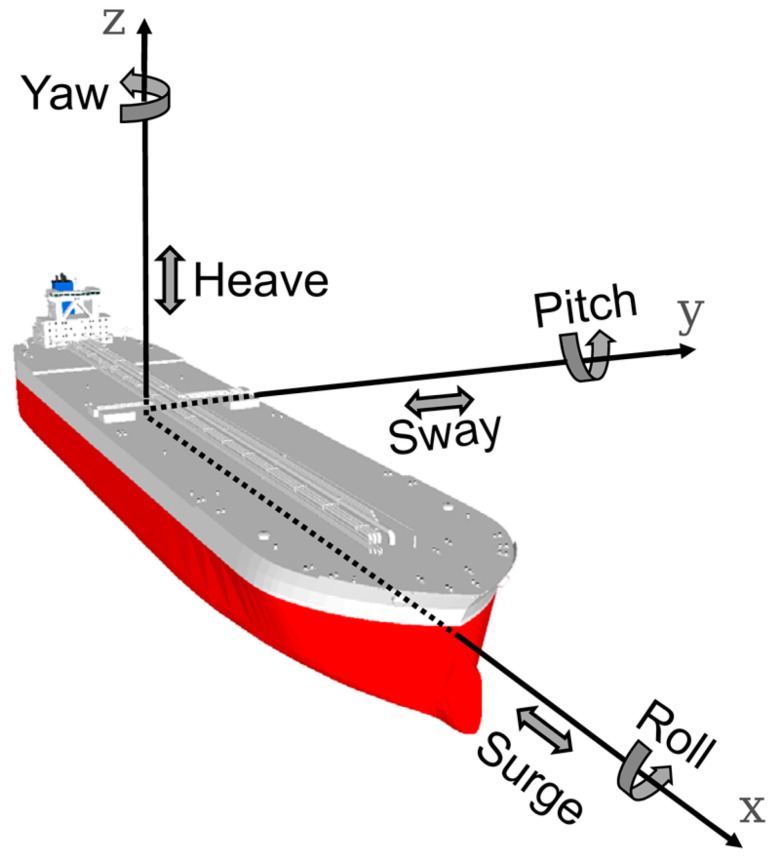
Vessel six degrees of freedom (after [65]).

The second type of spatial change, vessel motion, is caused by influence of hydrostatic, hydrodynamic, and wave forces. It includes the six main degrees of freedom (heaving, swaying, surging, yawing, rolling and pitching—see Figure 5) and, as shown by the observations regarding antenna alignment, should also be included in the analysis [64].

Equation set describing relations between the forces and their impact on vessel motion is presented in Equation (9)

(9) $$\begin{array}{cc} m\ddot{x} & =F_{x}\left(t\right)=X_{r}\left(t\right)+X_{\omega }\left(t\right)+X_{f}\left(t\right)\\ m\ddot{y} & =F_{y}\left(t\right)=Y_{r}\left(t\right)+Y_{\omega }\left(t\right)+Y_{f}\left(t\right)\\ m\ddot{z} & =F_{z}\left(t\right)=Z_{r}\left(t\right)+Z_{\omega }\left(t\right)+Z_{f}\left(t\right)\\ I_{G_{xx}}\ddot{\psi } & =M_{x}\left(t\right)=K_{r}\left(t\right)+K_{\omega }\left(t\right)+K_{f}\left(t\right)\\ I_{G_{yy}}\ddot{\theta } & =M_{y}\left(t\right)=P_{r}\left(t\right)+P_{\omega }\left(t\right)+P_{f}\left(t\right)\\ I_{G_{zz}}\ddot{\phi } & =M_{z}\left(t\right)=R_{r}\left(t\right)+R_{\omega }\left(t\right)+R_{f}\left(t\right) \end{array}$$

where *m* is the mass of the analyzed vessel, $I_{G_{xx}}$, $I_{G_{yy}}$, $I_{G_{zz}}$ are moments of inertia from the xx, yy, zz directions, respectively. Symbols *x*, *y*, *z* specify the position with respect to the axes, and ψ, θ, ϕ are angular measures for roll, pitch, yaw motions, respectively. *X*, *Y*, *Z* are the component forces acting along the x, y, z axes, respectively—resulting from hydrostatic, hydrodynamic and wave-related forces, respectively, defined by the footnotes *r*, *w*, *f*. Similarly, *K*, *P*, *R* are the moments of forces in the xx, yy, zz directions defined by the same footnotes. As a result, in the presented approach vessel motions are described in six degrees of freedom in accordance with Newton’s laws written for each direction.

Described phenomena related to motions of the vessel are among the major factors distinguishing maritime networks from on-shore communication systems (like cellular networks, radio links or vehicular networks), and so should be an element of the environment modeling included in a research process.

Solving a system of the above differential equations in a computationally efficient manner is a difficult task and a subject of numerous papers [66,67,68], as apart from its impact on maritime radio communication, it is a very important factor in ship design, analysis of cargo movement and, in its simplified version, in visualizations for training simulations. However, due to the complexity of the problem, often the computational resources at our disposal are insufficient to find a solution within the given time frame and it is necessary to adopt various simplifications.

One of techniques proposed to simplify the task is called 2d + t [69] and proposes a decomposition of a complex system of equations describing motion in 3D space into a number of simpler problems in two-dimensional space. In this case each surface crossing a ship hull is analyzed step by step [70]. Even after applying this simplification, the analysis of ship motion will be strongly linked to a spatial model that takes into account the dimensions and mass distribution of a particular vessel.

Another solution aims to map the effect of wave motion on wireless communications while maintaining computational efficiency, by modelling the pitch motion as a sum of trigonometric functions with different periods [54]. This solution is of particular interest in our case, as it provides a good accuracy for systems using high-gain omnidirectional antennas with a narrow vertical beam of a few degrees, which are commonly used in practice [6,54,55].

## 5. Proposed Wireless Link Selection Based on Deep Learning

The presented analysis of the propagation aspects of maritime wireless communication clearly shows why an attempt to evaluate TCP session performance using low-level parameters or even measured RTT and PER metrics of the network layer of the link can be very difficult. Although many numerical TCP models have been proposed [71,72,73], modeling link throughput using analytical formulas seems to be unreliable in real-word systems [74] due to a large number of parameters affecting this throughput. Moreover, since obtaining values of physical parameters comprehensively describing a wireless-link is difficult, the use of highly detailed analysis methods (which account for, e.g., dynamically changing TCP packet loss ratio [75]) become hard to implement in practical cases [76].

Even use of the active TCP testing is of limited utility, as while it directly measures TCP throughput of a particular link, the generated test traffic periodically consumes all available communication resources. In case of maritime communication, where we are interested in establishing communication over relatively long distances and as a result available throughput is limited, this method of active testing should be used with caution—as rarely as possible and for shortest possible periods. Such approach in turn limits the quality of resulting link assessment in the described, highly dynamic environment [77].

To further complicate the situation, the access environment which we want to use for coastal maritime communication is a heterogeneous one. Recent and ongoing research indicates [5,6,78,79,80], that a heterogeneous approach would be able to meet the requirements of maritime communication in near future [81], by integrating various already available, well-tested wireless communication techniques [6]. An example of such a system is presented in [6] and utilizes node mobility management mechanisms and multi-hop mesh network—however its efficiency is still dependant on the correct operation of link selection algorithms.

In this situation we would like to propose a solution providing a TCP throughput estimation of quality similar to that provided by the active TCP measurements, but with much lower traffic overhead (comparable to PER or RTT tests). Such an approach will allow us to retain advantages of making handover decision (including link selection) based on TCP throughput parameter (a direct, intuitive value directly describing the amount of communication resources available for applications), while minimizing resource consumption associated with the necessary measurements. The solution is based on supervised deep learning algorithms which are widely and successfully used as solutions for problems requiring analysis of complex data sets [82]. As deep learning brings beneficial results in a number of applications [83], especially when analytical models (most often do to high number of parameters) are barely computable and hard to implement, it seems logical to apply it in our case.

From many different uses of deep learning we have concentrated on two specific supervised learning tasks: regression and classification [84]. By analyzing neural network structures and learning procedures used in each of the above cases, we have designed two different solutions for our link selection problem: Deep Learning Regression Link Evaluation Method (DLR-LEM) and Simultaneous Deep Learning Link Evaluation Method (SDL-LEM).

**Figure 6:**
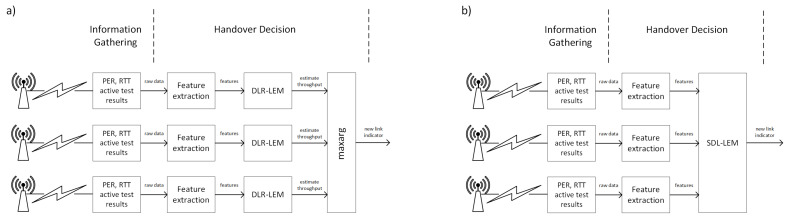
Link selection procedure according to: (**a**) DLR-ESM method, (**b**) SDL-LEM method.

In Deep Learning Regression Link Evaluation Method the link-selection problem is represented as a regression task in which each link is evaluated separately and the evaluation results are compared to find the best one. The link selection procedure performed in accordance with this method is presented in Figure 6a). As we are interested in obtaining a TCP throughput prediction, the output is an estimation of TCP connection throughput based on given features. With TCP connection throughput estimated independently for each of the links, the selection algorithm is similar to the one used in a simple, active TCP testing approach. However, this time the estimated values of the throughput, instead of directly measured ones, are used as link metrics.

In case of Simultaneous Deep Learning Link Evaluation Method, features of all links are evaluated simultaneously. In this case a softmax layer is used for converting the outputs of dense layers into a probability distribution of a number of possible outcomes [85]. In our approach *I* classes are defined, each representing one of the available communication links. As a result, the network does not provide an estimation of TCP throughput, but directly indicates the best link based on input information describing all available links, by classifying it as an output class representing a specific link (see Figure 6b).

### 5.1. Data Collection and Processing

Since in the proposed solutions we are taking advantage of the ability to generalize relations between input features and target outputs offered by deep learning methods, each evaluated link must be described by a set of parameters. To obtain such parameters, we have chosen to employ previously described testing methods, which are characterized by a low amount of test traffic they require—PER and RTT. We use two types of messages: PER testing messages and round-trip-time testing messages (RTT) and enable us to evaluate link losses and latency—two important factors impacting TCP throughput [41].

RTT and PER tests are performed independently and their results are collected also separately, pending further processing. Both RTT and PER active tests are performed between a vessel and a base station by means of dedicated messages, allowing us to measure wireless link characteristic at network layer.

RTT test works in a way similar to the well-know ping service—the application sends a request every $t_{RTT}$ and measures time that elapsed until it receives a response. The results of RTT test give explicit delay values and they are directly used to form a raw data vector.

**Figure 7:**
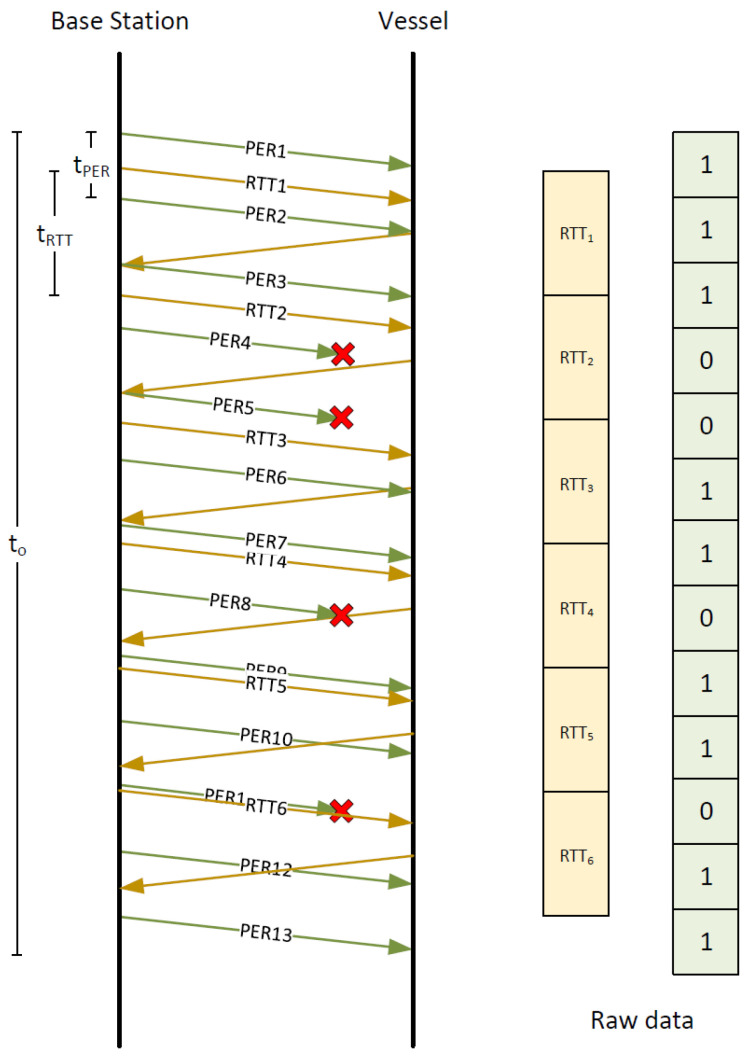
Raw data collecting process.

During PER test, a group of messages is sent from a base station with interval of $t_{PER}$ between subsequent messages. Each of messages is marked with an unique sequence number, so a receiving mobile node is able to detect a message loss. The result of the test is a raw data vector in which each received PER message is represented as 1, while a lost message is represented as 0. Figure 7 illustrates this process with an example in which messages PER4, PER5, PER8, PER11 are lost in transmission (which is indicated by red cross marks).

**Figure 8:**
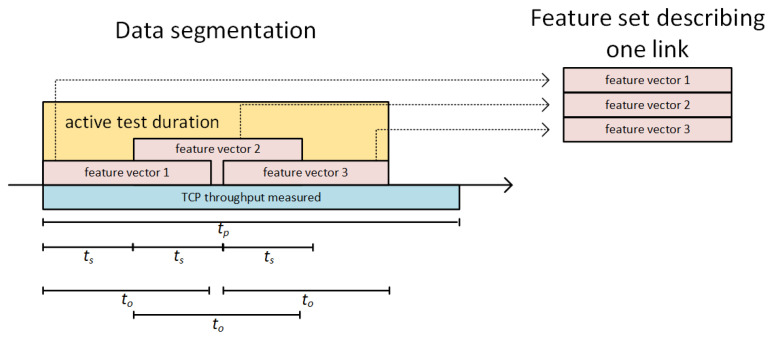
A segmentation procedure with illustration of its parameters ($t_{o}$, $t_{s}$) resulting in obtaining of 3 segments (*M* = 3).

To make proposed methods more responsive to changes of the link quality, we decided to introduce a data segmentation procedure. This procedure allows us to increase the number of parameters describing wireless links. Instead of using raw data vectors (containing direct results of PER and RTT tests) as inputs for a neural network, the vectors are first treated as time-series and than divided into time segments (see Figure 8), to allow calculation of a number of their statistical parameters for use as feature sets serving as inputs for the neural network. The time length of each segment is denoted as $t_{o}$ and the time difference between starts of subsequent segments is marked as $t_{s}$. As a result, *M* segments are obtained from a given set of raw data vectors gathered during the active RTT and PER tests. Features extracted from raw data vectors generated by RTT tests include: length, sum of elements, maximum values, minimum values, mean, variance, skewness, kurtosis and every tenth percentile. Raw data vectors of PER test are used to calculate feature vectors containing the following features: length, number of zeros, sum of elements, mean, variance for PER raw data.

As the deep learning network training procedure requires each network input to be assigned an expected output, a TCP throughput is measured for each link over time $t_{p}$. For DLR-LEM method, which is expected to provide TCP throughput estimations this value is directly used as an output in a learning process. In case of SDL-LEM method, a hot-one encoding is used to prepare a learning output value—measured values of TCP throughput for all links are compared and the one of the highest one is marked with a value of “1” in the output vector, while remaining links are marked with value of “0”.

### 5.2. Network Structures

The selection of neural network structures which we employ in our proposed solutions was made on the basis of literature studies. Following a number of researches (e.g., [50,86,87,88]) related to the use of deep learning in wireless networks, we decided to utilize network structures employing both Long Short-Term Memory (LSTM) [89] and Dense neurons layers [90].

Specific network structures designed for a particular method (i.e., DLR-LEM and SDL-LEM) are different, as each needs to address a different task adapted as a core of our solution: a regression task for DLR-LEM, and a classification task in case of SDL-LEM.

**Figure 9:**
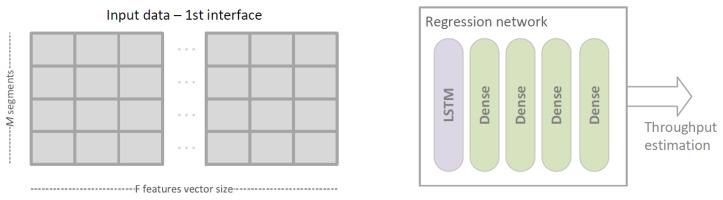
DLR-LEM network structure.

DLR-LEM network contains LSTM layer followed by a number of Dense layers. It takes data describing a single link as input and estimates predicted TCP throughput as output—see Figure 9.

**Figure 10:**
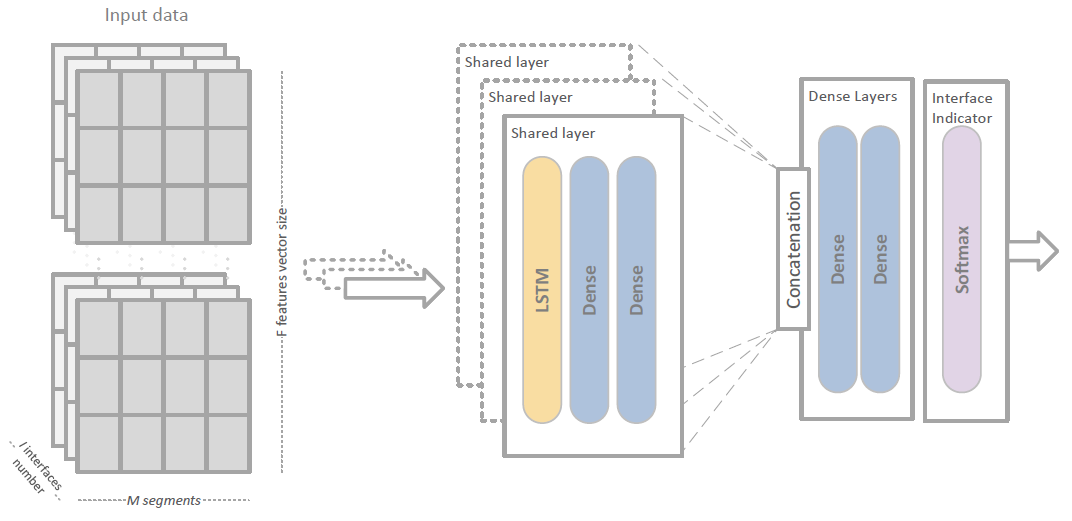
SDL-LEM network structure.

In case of the SDL-LEM approach (see Figure 10), the input contains information describing all links available to the mobile node (a three dimensional feature array—multiple values of multiple features for multiple interfaces). Initially, feature vectors for each link are processed by dedicated parts of the network (shared layers). To make the learning procedure more effective and link-order insensitive, a separate instance of the same structure is used for each link. Outputs of the instances are concatenated and are inputs for the further part of the model. Since in such an architecture input information regarding all the links can be used by all shared layers less data is required to train the model. After concatenation, a number of dense layers compare outputs of shared layers and indicate the best possible interface with use of a softmax layer.

Hyperparametrs of described structures have been examined in a tuning procedure including evacuation of different numbers of stacked layers, kernel sizes and optimization algorithms. Results of this work summarizing hyperparametrs sets for both DLR-LEM and SDL-LEM are presented in Table 1. During the training procedure, a model checkpoint have been saved in 50-epoch intervals and analyzed later to avoid overfitting and find parameters providing best performance. Duration of the training varied depending on evaluated network structures and the value of *I* parameter. The process took as long as few hours for the SDL-LEM method and *I* = 7, which is a considerable time. However, we must remember, that in practice the learning process occurs relatively infrequently—e.g., once a day port authority prepares and distributes ready-to-use neural networks to interested vessels. Much more important for practical applications is the time required to perform the link selection process itself, which in case of proposed solution was lower than 25 ms (computing on standard computer with Intel Core i7-8750H CPU and NVIDIA GTX 1050 Ti), which is incomparably smaller than $t_{o}$.

## 6. System Modelling and Evaluation

Since the proposed deep learning solutions require data input both during the learning phase and later when used to support handover decisions, in the absence of sufficient real-world measurement data, we have decided to use simulation methods for a dual purpose:

- the learning phase of neural networks and selection of their hyperparameters,
- verification of results provided by the employed networks.

**Figure 11:**
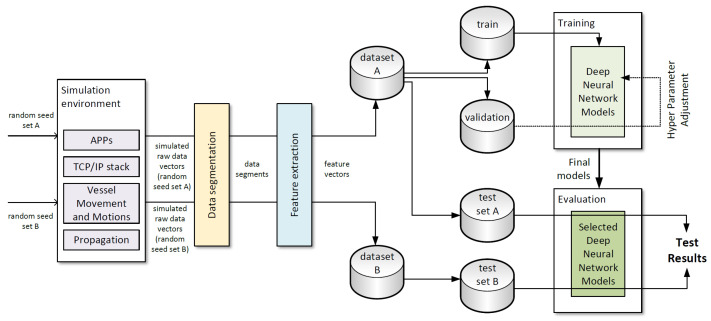
Experiment methodology.

Our simulation environment (described below) is employed to provide measurement data (see Figure 11) and includes the elements crucial for precise modeling of maritime communication environment described in Section 4. As already indicated, the higher that commonly employed complexity of the model is of special importance in this case, as we intend to verify the operation of the deep learning-based solutions in an environment closely resembling actual real-world conditions, to justify their use in place of classical methods based on deterministic calculations.

The simulation model has been implemented in Omnet++ simulator [93] with INET extension [94] which required significant enhancements to its standard functionality to provide appropriate models of radio signal propagation and vessels movement/motions.

**Figure 12:**
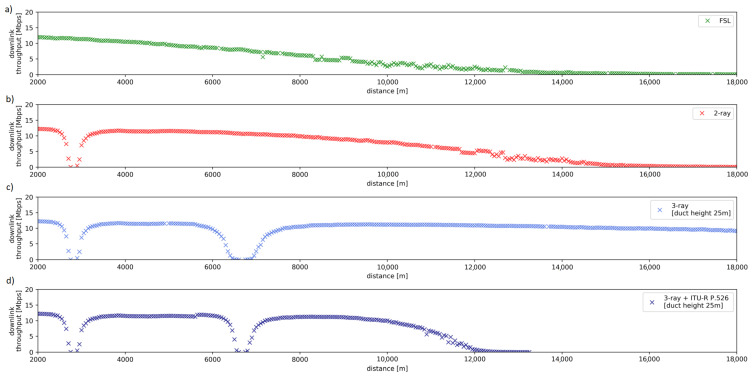
The average TCP throughput for different propagation models.

The impact of an appropriate propagation model selection can be seen in Figure 12, where we present values of average TCP connection throughput as a function of distance between communicating nodes, measured in the simulated environment with different propagation models employed. Subsequent plots from (a) to (d) refer to Free Space Loss (FSL) model, two-ray model, three-ray model (evaporation duct height 25 m) and three-ray model with diffraction calculated according to Equations (5). Literature study indicates that FSL and two-ray models do not fit deep nulls observed in data sets obtained in real-world measurement campaigns [52]. We also observe that use of the three-ray model without taking into account diffraction effects is reasonable only for relatively short distance, because of the third ray that significantly decreases overall path loss. Without diffraction modeling included, it results in excessively high throughput of the simulated TCP connection (see Figure 12c) at long communication ranges. Based on the above observations, we have chosen to employ the three-ray model extended to include diffraction effects in our experiments, as it provides results best matching a real-world environment [95].

To provide sufficient measurement data for learning and evaluation of proposed solutions, a 350 simulation scenarios have been conducted using different random seed values. During each scenario parameters have been randomly chosen using ranges and distributions indicated in Table 2. Each simulation lasted 2020 s of simulated time with a mobile node moving within a 10 km × 95 km area. Observing historical AIS data describing vessel movement along shipping lanes, we decided to use an enhanced Linear Mobility Model from the INET framework to model movement of vessels arriving and departing ports and moving along shipping lanes.

As far as precise motion modelling is concerned, we have observed that solving the equation set (9) fully describing motion of a maritime vessel is time consuming and its solution is always related to a particular shape of a vessel, as well as its weigh distribution. In this situation, in an attempt to make results more general while still retaining a high degree accuracy of the simulation, we have decided to limit the motion modelling to pitch motion, as it is the one that has the greatest impact on maritime wireless communication [54,96]. For this purpose, a randomly selected $\Theta_{max}$ is generated for each scenario and used to model pitch changes of the vessel as described by Equation (10).

(10) $$\begin{array}{c} \Theta \left(t\right)=\sin\left(\frac{2\pi }{t_{w}}+\frac{2\pi }{4t_{w}}\zeta_{\phi }t+\varphi_{\Theta_{1}}\right)+\sin\left(\frac{2\pi }{16}t+\varphi_{\Theta_{2}}\right)) \end{array}$$

It is an arbitrary function with random parameters: wave period—$t_{w}$, pitch change factor—$\zeta_{\phi }$, staring points $\varphi_{\Theta_{2}}$ and $\varphi_{\Theta_{2}}$. As a result pitch motions can be modeled, often introducing notable changes in communication quality. To bring the simulation closer to the reality, commercial off-the-shelf antennas characteristics have been used—in the base station, a sector antenna with a 19 dBi gain and sector width of 120${}^{\circ }$ [59] and on the vessel, an omnidirectional antenna with a 10 dBi gain and vertical beamwidth of 10${}^{\circ }$ [60].

To obtain the measurement data required by the proposed solutions, an application collecting RTT and PER metrics at network layer has been implemented in the simulated environment, while measurements of actual TCP throughput were conducted using standard INET traffic source and sink models.

For each simulation scenario two runs have been conducted using the same random seed and parameter values. During the first a TCP connection between the mobile node and a onshore network server has been established and its throughput measured. During the second, the application for RTT/PER measurements has been used to obtain delay/packet loss measurements. Such an approach allows us to collect data regarding both actual (simulated) TCP throughput, and results of RTT/PER testing in the same situation scenario (as both runs use the same random seed and parameters) without the two active processes impacting each other (they are employed during different simulation runs). As a result, we are able to obtain parameters intended to be used as input data for neural networks and their corresponding TCP throughput values which can be used in learning, validation and verification processes.

Of all 350 conducted simulation scenarios, results of 300 have been used as a training, validation and testing data (Dataset A) while results of remaining 50—as a separate data set used only for testing (Dataset B).

As presented in Figure 8, raw data vectors containing results of RTT/PER measurements were divided into segments. Each of the segments was then unambiguously assigned to a training, validation or testing set, so there was no possibility that the same feature vectors obtained from were used for both training and testing. However, in case of Dataset A, it is possible for data segments obtained in raw vector segmentation process to overlap ($t_{s}$ < $t_{o}$ and *M* > 1), which might lead to some some elements of raw data vectors being used in both training and testing sets (see Figure 11). Such a method allows us to test the proposed solution in an environment very similar to the one which has been used for its training, while not making a mistake of using the same input information in both learning and testing phases of neural network operation. Additionally, to verify the proposed method in an environment different from the one from which the learning data has been obtained, Dataset B was employed exclusively in the testing phase.

### 6.1. Evaluation Metrics

Although link quality assessment and link selection algorithms have been a well-known research subject for some time now, there is no standardized and commonly accepted way to evaluate and compare different proposed solutions. However, five main aspects of evaluation have been recommended in the literature: reliability, adaptability, stability, computation cost and probing overhead [38]. Based on this recommendation, we use three numerical metrics defined further in this section to directly address evaluation of reliability and probing overhead. Moreover aspects of adaptability and stability are included in the evaluation by employing multiple data sets generated by the simulation scenarios conducted with different random seed values.

The most intuitive way to compare selection algorithms is to use an accuracy metric, which can be defined by Equation (11):

(11) $$\eta_{A}=\frac{\sum_{n=1}^{N}a_{n}}{N}$$

where $a_{n}$ represents the result of the *n*-th of *N* simulation scenarios, each characterized by a specific random seed. It equals 1 if the best possible link has been chosen and 0 otherwise.

This metric is a relatively simple one, directly showing the ratio between a number of correct predictions and a total number of all predictions. However, when applied in a real-world scenario, it does not produce results close to an actual user experience. Missing the selection of the best link always impacts the metric in the same way, regardless of what its actual impact on the user experience is—it does not matter if the choice results in only slightly lowered or severely degraded performance. For this reason, we have decided to employ more application-oriented metrics in our evaluation.

The first is a normalized throughput presenting ratio between throughput of the selected link and the throughput of the best link available—see Equation (12).

(12) $$\eta_{T}=\frac{\sum_{n=1}^{N}\frac{T_{i_{s}}\left(t_{p}\right)}{{max}_{i}\left(T_{i}\left(t_{p}\right)\right)}}{N}$$

where $T_{i}\left(t_{p}\right)$ is a TCP throughput observed over time $t_{p}$ for *i*-th link, and $T_{i}s\left(t_{p}\right)$ is a similar parameter for the link selected by the evaluated solution. The second (see Equation (13)) additionally includes information about the amount of test traffic transferred over wireless links that was required to accomplished the selection procedure. Thus, in this case, an impact of probing overhead can be evaluated and compared to reference methods.

(13) $$\eta_{B}=\frac{\eta_{T}}{\sum_{i}D_{i}\left(t_{o}\right)}$$

where $D_{i}$ in a number of bits being sent over the *i*-th interface in order to perform link evaluation with respect to particular method. It should be noted that $\eta_{T}$ is a metric representing method efficiency observed in a longer perspective (over $t_{p}$). However active test transmissions occur only in $t_{o}$.

### 6.2. Results and Discussion

To verify the proposed link-selection solutions, they were implemented using the Keras tool [97] (a high level framework for deep learning applications). The supervised learning process was performed using a training dataset derived from Dataset A, and than a validation data set (also based on Dataset A) was employed to find a set of hyperparameters providing the best results (see Figure 11). After such preparation, both proposed methods were tested using test data obtained from Dataset A (which simulated employed of the solution in a situation similar to its learning environment) and Dataset B (which allowed them to be verified in an environment of possibly different characteristics). Output of the method under test for a given input set of feature vectors, was compared to the corresponding values of TCP throughput measured in a simulation environment (for DLR-LEM) or to a link correctly selected based on such values (in case of SDL-LEM).

The results obtained for the proposed methods were compared to the classic link-selection methods directly utilizing active RTT, PER and TCP measurements. Each of the analyzed scenarios was repeated N = 500 times, and the average values of the presented results were determined taking into account the 95% confidence interval.

In Table 3 and Table 4, accuracy ($\eta_{A}$) and normalized throughput ($\eta_{T}$) results for a classic link-selection methods directly utilizing results of active PER, RTT and TCP measurements (described in Section 3) are presented. The results were obtained for a different number of links, different values of $t_{o}$ and $t_{p}$ = 600 s. Analyzing the tables, it can be seen that only active TCP measurement offers acceptable results. For all values of $t_{o}$, PER and RTT methods perform relatively poorly with $\eta_{A}$ between 0.07 (for RTT method and seven interfaces, $t_{o}=3$) to 0.7 (PER method, two interfaces and $t_{o}=30$) which corresponds to $\eta_{T}$ between 0.21 and 0.8, respectively. It can also be seen, that performance of both RTT and PER methods decreases with increasing number of links to choose from. In contrast, the active TCP measurement method provides better results and is not so sensitive to the number of links under consideration, because the real value of available throughput is measured for each of available links. As expected, the results also confirm that the longer observation period is, the better results can be expected.

It is evident, that the results of TCP active test link selection method are promising as it obtained average values of $\eta_{T}$ between 0.74 and 0.88. Unfortunately, a volume of test traffic required to obtain such satisfying results is unacceptably high, which is clearly shown by values of bit efficiency ($\eta_{B}$) presented in Table 5. It should be noted that bit efficiency ($\eta_{B}$) values for the active TCP-based method two or three orders of magnitude smaller than in case of RTT and PER-based solutions.

After obtaining the reference results on classical methods used in the link selection process, we performed a series of experiments intended to verify accuracy of the proposed methods designed to obtain results comparable to a direct TCP measurement, while keeping the traffic overhead similar to that sufficient for PER and RTT tests.

**Figure 13:**
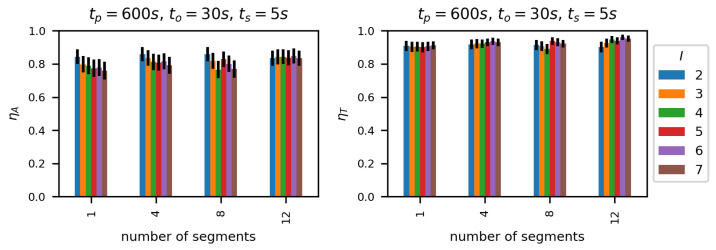
Number of segments impact on *η_A_* and *η_T_* metrics—DLR-LEM.

**Figure 14:**
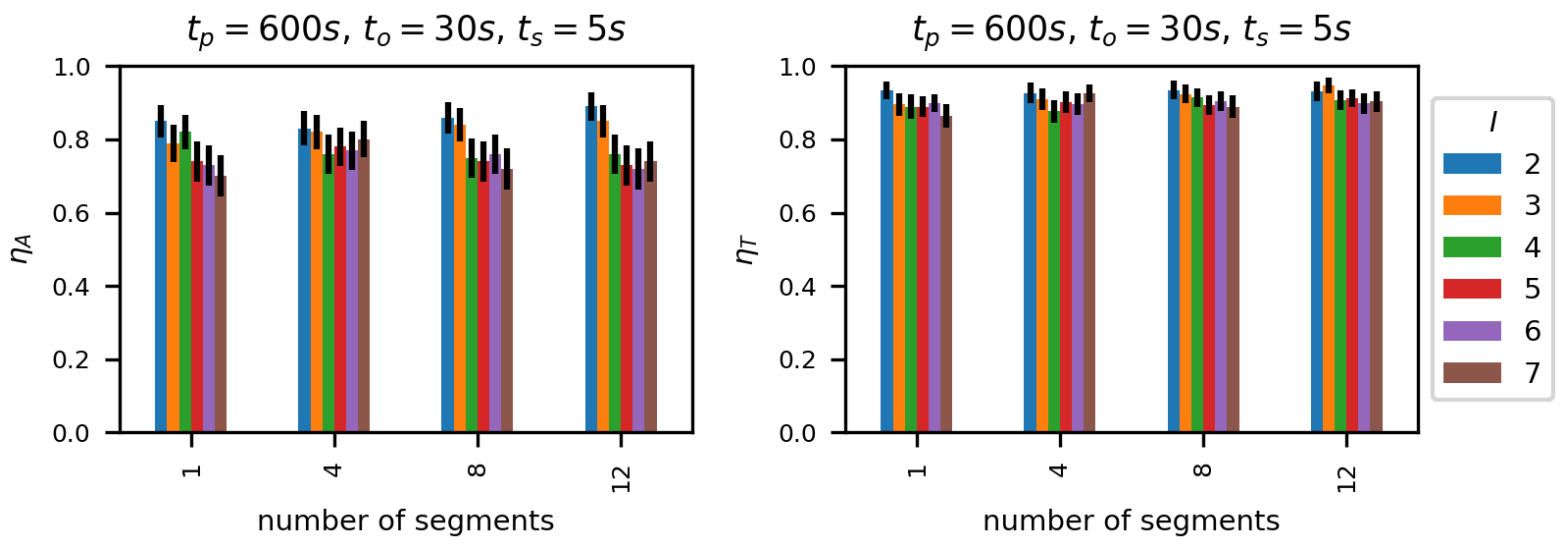
Number of segments impact on *η_A_* and *η_T_* metrics—SDL-LEM.

Figure 13 and Figure 14 present efficiency of the proposed methods showing $\eta_{A}$ and $\eta_{T}$ values, respectively, for DLR-LEM and SDL-LEM solutions. To make the results comparable with the best results of previously mentioned methods, the following values of link selection method parameters were selected: $t_{p}$ = 600 s, $t_{s}$ = 5 s and $t_{o}$ = 30 s. A test dataset derived from Dataset A has been employed. It should be noted, such a test dataset is, as described in Section 5.1, not identical to learning and validation datasets, despite being derived from the same simulated measurement results. Additionally, the results are obtained for different values of *M* – the number of segments describing a link being evaluated (see Figure 8). With greater number of the segments more data describing a wireless link is used in the assessment process increasing the possibility to incorporate a wide scope of communication environment events and changes, especially changes occurring less frequently.

We can see that normalized throughput ($\eta_{T}$) values are relatively high, between 0.9 and 0.98 and increase with the number of segments *M*. Moreover, the number of links under consideration has a very small impact on $\eta_{T}$—similar to the impact of this parameter on TCP active test results.

**Figure 15:**
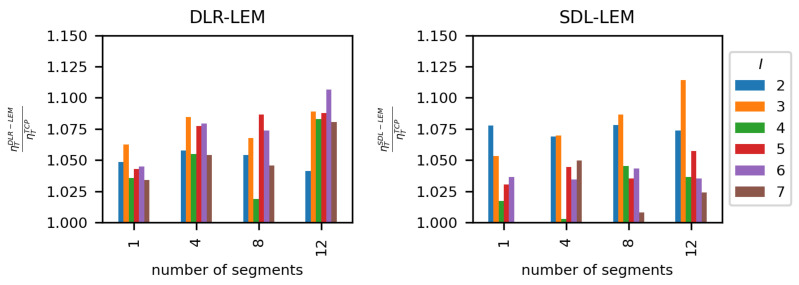
$\eta_{T}$ Relation between active TCP test approach and proposed methods.

A comparison of $\eta_{T}$ evaluation of active TCP measurement method and the proposed solutions is presented in Figure 15. It can be seen, that for all analyzed parameter values (number of links, and number of segments) proposed methods perform better than the best of the classic solutions, in case of SDL-LEM M = 12 reaching a 12.5% advantage.

Having verified that proposed methods provide accurate results when tested in an environment similar to the one used in the learning phase, we have conducted further experiments to verify their adaptability and stability. For this purpose, we conducted additional evaluations, using test datasets obtained from Dataset A and Dataset B separately. Dataset A, based on the same results as learning and validation datasets, allows us to verify operation of our solutions in conditions similar to their learning environment. The use of Dataset B makes it possible to verify the solutions in conditions different from conditions used in their learning process.

**Figure 16:**
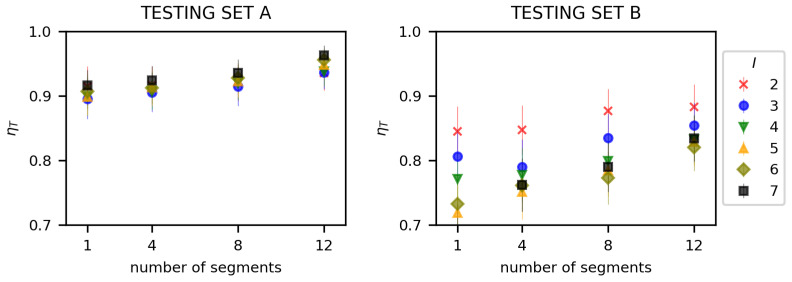
Number of segments impact on selection efficiency for Dataset A and Dataset B—DLR-LEM.

**Figure 17:**
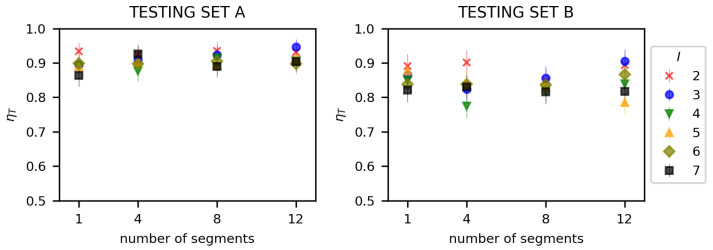
Number of segments impact on selection efficiency for Dataset A and Dataset B—SDL-LEM.

Obtained results clearly indicate (see Figure 16 and Figure 17), that in case of Dataset A, the approach using deep learning regression (DLR-LEM) brings better results than the method formulated as a classification problem (SDL-LEM). However, in case of Dataset B, results indicate advantage of classification approach. Thus, usage of the method employing deep learning regression can be recommended for environments similar to scenarios used during the learning procedure and method formulated as a classification problem should be used otherwise.

**Figure 18:**
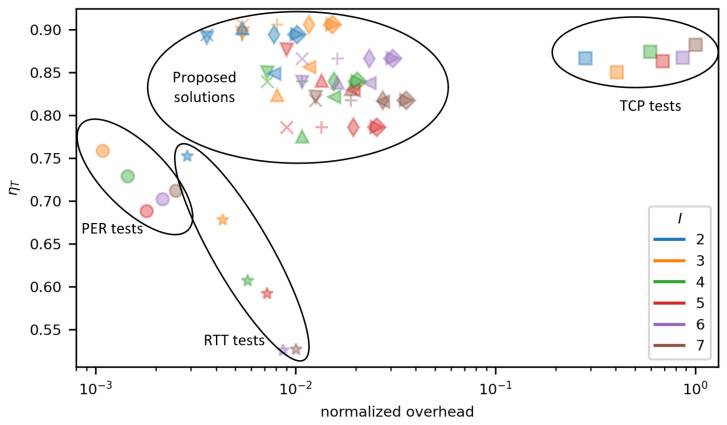
$\eta_{T}$ over overhead for evaluated methods: ■—TCP; ★—RTT; •—PER; ×—DLR-LEM M1; +—DLR-LEM M4; ◊—DLR-LEM M8; ⋄—DLR-LEM M12; ▽—SDL-LEM M1; △—SDL-LEM M4; ◃—SDL-LEM M8; ▹—SDL-LEM M12.

Presented results show varied, but superior ability of proposed methods to select a link preferred in terms of the expected TCP throughput, compared to the most popular classic solutions used as reference. Additionally, we should take into account, that the proposed solutions utilize low overhead RTT/PER measurements instead of an actual TCP throughput testing. In order to show the benefits of the proposed methods in terms of their smaller overhead, a plot showing the relationship between $\eta_{T}$ and the normalized overhead has been presented in Figure 18.

The results visible in Figure 18 form four distinct groups. The first and second groups consist of results achieved by RTT (★) and PER (•) method. They are located in the lower left corner of the plot which indicates, that they are characterized by the lowest traffic overhead, but also offer unacceptably low values of $\eta_{T}$. PER-based method generally performs better than RTT-based, both in terms of accuracy of higher $\eta_{T}$ and lower overhead. It should be noted, that the above results confirm characteristics of RTT and PER methods described in Section 3. The third group is formed by results of the direct TCP measurement method and is located in the right-hand side of the plot signifying that relatively high $\eta_{T}$ values were achieved at a cost of a very high traffic overhead. The fourth group represents results of the proposed methods and its location clearly highlights their advantages—very high values of $\eta_{T}$ (including the highest for all methods under test) at a relatively low traffic overhead (in this case the overhead is approximately a dozen times lower than in case of the direct TCP measurement method).

## 7. Future Works

With the presented, simulation-based evaluation of the proposed solution showing promising results, a natural direction of future works is to verify the efficiency of the proposed solutions in real-world experiments. Despite our care to prepare an advanced simulation environment taking into account a number of important aspects of wireless propagation in maritime communication, there might still be a need for adjustments by means of additional training performed in accordance with a learning transfer methodology [98,99] to adapt our models to its intended environment and wireless technologies.

Moreover, the use of deep learning solutions requires great amount of input data during a training phase. Since our solution assumed that training procedures are performed in a centralized system, to make the proposed approach more applicable in general use-cases, it would be beneficial to enhance out solution with federated learning mechanisms [100] giving the possibility to generates a global model through the collaboration between different companies or authorities without the need for sharing raw input data (which is often considered confidential).

## 8. Conclusions

The analysis presented in the paper have shown, that basic decision methods operating with a low traffic overhead (such as direct use of measured PER or RTT values) provide unsatisfactory results. On the other hand, methods like active TCP throughput measurement introduce a significant traffic overhead and, as a result, waste limited communication and energy resources. To counteract the limitations of such basic methods, we have proposed two data analysis and decision making solutions employing deep neural networks.

The proposed solutions aim to retain advantages of the PER/RTT classic methods, by employing essentially the same, low traffic overhead while performing measurement of link parameters. The information is then processed using natural networks designed to use their ability to perform regression and classification tasks. As efficiency of neural networks depends on their training phase and the data used in that process, we decided to devote a special care to obtain such a reliable data. For that purpose, we have analyzed and described a number of important aspects affecting radio propagation over sea areas, and developed an especially modified simulation model as a measurement source.

The results obtained during the evaluation show that by applying the proposed methods it was possible to maintain (and sometimes surpass) the quality of link evaluation provided by direct TCP measurement, while reducing the required test traffic by about two orders of magnitude. Such a low impact on the communication system, combined with high accuracy, acceptable time of learning and fast response of the trained neural network, make the proposed methods an useful solution for a heterogeneous, coastal maritime communication environment.

## Figures and Tables

**Table 1:** Neural network structure and training details.

	DLR-LEM	SDL-ELM
Network structure *	$L_{64}2xD_{64}3xD_{16}D_{8}D_{1}$	${\left[L_{64}D_{64}5xD_{32}\right]}_{I}D_{32}D_{32}D_{I}$
Activation (except the last layer)	ReLU	ReLU
Activation (the last layer)	softplus	softmax
Kernel initializer	He uniform [91]	He uniform [91]
Loss function	MSE	Categorical Crossentropy
Number of epochs	6000	6000
Checkpoint frequency	50	50
Batch size	512	512
Optimizer	Adam [92]	Adam [92]
Learning rate	0.01	0.01

* *L* and *D* stand for LSTM and Dense layers, respectively. Number in subscription refers to number of neurons in each layer.

**Table 2:** Simulation Parameters.

Parameter	Value
Warmup period	20 s
Initial distance	1–20 km
vessel speed	3–10 mps
Max pitch	2${}^{\circ }$–10${}^{\circ }$
Radio	IEEE 802.11ac
Frequency	5.8 GHz
Channel Bandwidth	20 MHz
Tx Power	30 dBm
BS antenna gain	19 dBi
Vessel antenna gain	10 dBi
Antennas height	7 m
Evaporation duct height	30 m
$\zeta_{\Theta }$	0–1
$\varphi_{\Theta_{1}}$,$\varphi_{\Theta_{1}}$	0${}^{\circ }$–180${}^{\circ }$
$t_{w}$	3–15 s
$t_{PER}$	0.2 s
$t_{RTT}$	0.5 s

**Table 3:** $\eta_{A}$ results.

	*I*	2	3	4	5	6	7
	$t_{o}$						
PER	3	0.62	0.50	0.39	0.39	0.35	0.31
	9	0.68	0.55	0.46	0.45	0.41	0.37
	15	0.68	0.58	0.47	0.46	0.48	0.43
	21	0.69	0.58	0.56	0.51	0.47	0.49
	30	0.70	0.59	0.56	0.53	0.47	0.51
RTT	3	0.53	0.29	0.21	0.18	0.09	0.07
	9	0.61	0.49	0.37	0.29	0.27	0.22
	15	0.68	0.51	0.43	0.36	0.30	0.27
	21	0.66	0.53	0.46	0.34	0.33	0.30
	30	0.66	0.53	0.44	0.40	0.35	0.31
TCP	3	0.69	0.60	0.58	0.58	0.56	0.54
	9	0.76	0.65	0.61	0.62	0.63	0.62
	15	0.75	0.69	0.67	0.65	0.66	0.68
	21	0.78	0.72	0.70	0.71	0.67	0.68
	30	0.79	0.73	0.74	0.69	0.72	0.72

**Table 4:** $\eta_{T}$ results.

	*I*	2	3	4	5	6	7
	$t_{o}$						
PER	3	0.72	0.64	0.56	0.57	0.54	0.50
	9	0.77	0.71	0.63	0.62	0.60	0.58
	15	0.77	0.72	0.65	0.64	0.67	0.63
	21	0.79	0.71	0.72	0.68	0.67	0.67
	30	0.80	0.72	0.72	0.71	0.68	0.70
RTT	3	0.64	0.44	0.37	0.33	0.24	0.21
	9	0.72	0.63	0.55	0.47	0.45	0.40
	15	0.77	0.66	0.60	0.54	0.50	0.46
	21	0.75	0.68	0.63	0.54	0.51	0.49
	30	0.75	0.67	0.62	0.59	0.55	0.49
TCP	3	0.78	0.74	0.75	0.77	0.75	0.74
	9	0.83	0.78	0.78	0.81	0.80	0.82
	15	0.83	0.81	0.82	0.83	0.85	0.84
	21	0.84	0.84	0.84	0.86	0.83	0.87
	30	0.87	0.85	0.87	0.86	0.87	0.88

**Table 5:** Bit efficiency $\eta_{B}$.

Test Name	2	3	4	5	6	7
TCP	9.26 × 10${}^{-9}$	6.33 × 10${}^{-9}$	4.41 × 10${}^{-9}$	3.79 × 10${}^{-9}$	3.02 × 10${}^{-9}$	2.65 × 10${}^{-9}$
RTT	7.84 × 10${}^{-7}$	4.70 × 10${}^{-7}$	3.16 × 10${}^{-7}$	2.46 × 10${}^{-7}$	1.82 × 10${}^{-7}$	1.57 × 10${}^{-7}$
PER	3.35 × 10${}^{-6}$	2.11 × 10${}^{-6}$	1.52 × 10${}^{-6}$	1.15 × 10${}^{-6}$	9.75 × 10${}^{-7}$	8.48 × 10${}^{-7}$

## Data Availability

Not applicable.
